# Opposing deer and caterpillar foraging preferences may prevent reductions in songbird prey biomass in historically overbrowsed forests

**DOI:** 10.1002/ece3.3497

**Published:** 2017-12-02

**Authors:** R. Keating Godfrey, Ellen H. Yerger, Timothy J. Nuttle

**Affiliations:** ^1^ Indiana University of Pennsylvania Indiana PA USA; ^2^ Present address: University of Arizona 1040 E 4th Street, PO Box 210077 Tucson AZ 85721 USA; ^3^ Present address: Civil & Environmental Consultants Inc. 333 Baldwin Road Pittsburgh PA 15205 USA

**Keywords:** Allegheny hardwoods, bottom‐up, caterpillars, Lepidoptera, Pennsylvania, tritrophic

## Abstract

Overbrowsing by ungulates decimates plant populations and reduces diversity in a variety of ecosystems, but the mechanisms by which changes to plant community composition influence other trophic levels are poorly understood. In addition to removal of avian nesting habitat, browsing is hypothesized to reduce bird density and diversity through reduction of insect prey on browse‐tolerant hosts left behind by deer. In this study, we excluded birds from branches of six tree species to quantify differences in songbird prey removal across trees that vary in deer browse preference. Early in the breeding season, birds preyed on caterpillars at levels proportional to their abundance on each host. Combining these data with tree species composition data from stands exposed to experimentally controlled deer densities over 30 years ago, we tested whether overbrowsing by white‐tailed deer reduces prey biomass long after deer densities are reduced. Our analysis predicts total prey availability in the canopy of regenerating forests is fairly robust to historic exposure to high deer densities, though distribution of prey available from host species changes dramatically. This predicted compensatory effect was unexpected and is driven by high prey abundance on a single host tree species avoided by browsing deer, *Prunus serotina*. Thus, while we confirm that prey abundance on host trees can act as a reliable predictor for relative prey availability, this study shows that quantifying prey abundance across host trees is essential to understanding how changes in tree species composition interact with ungulate browse preference to determine prey availability for songbirds.

## 1 INTRODUCTION

Growth in ungulate population densities, unchecked by the loss of natural predators and encouraged by expanding urbanization, has measurably altered temperate forest communities in many areas worldwide, including the Northeastern United States (Horsley, Stout, & DeCalesta, 2003), western Canada (Martin, Stockton, Allombert, & Gaston, 2010; J. Teichman, Nielsen, & Roland, 2013), central Japan (Nomiya et al., 2002), the Netherlands (Kuiters & Slim, 2002), and throughout northwestern Europe (reviewed by Hester, Edenius, Buttenschøn, & Kuiters, 2000). Decades of research and control programs have restored ungulate densities to levels comparable with those prior to the loss of large predators (Pennsylvania Game Commission 2017; Wallingford et al., 2015). However, it is evident that browsing preferences produce long‐term changes to plant community composition that disrupt ecosystems long after browsing pressure is reduced (Nuttle, Ristau, & Royo, 2014; Nuttle, Yerger, Stoleson, & Ristau, 2011).

In addition to direct effects on plant communities, overabundant ungulates indirectly disrupt populations of other herbivores (Wheatall, Nuttle, & Yerger, 2013) and predators of invertebrates such as songbirds (Nuttle et al., 2011). Bird species that rely on understory vegetation show the greatest loss in abundance and diversity (Allombert, Gaston, & Martin, 2005; DeCalesta, 1994; Martin et al., 2010; Mcshea & Rappole, 2000), and effects may persist decades after ungulates density is reduced (Nuttle et al., 2014). In a natural experiment on the effect of deer browse pressure on songbird abundance and diversity, Allombert et al. (2005) suggested that continent‐wide reductions in songbird populations across Europe could be due to the current and historic imprint of ungulates on the landscape, foresight of the now familiar, far‐reaching effects of trophic downgrading (Estes et al., 2011). Nearly, all of these studies suggest browsing reduces songbird abundance by direct removal of nesting habitat and decreases in food availability resulting from lower plant biomass. While several studies have shown drastic changes to understory plant composition, none have tested if variation in bird preference for or utilization of invertebrates across tree hosts may explain reductions in understory bird abundance in plots historically exposed to high browse pressure.

Classic predation studies show birds favor larger prey items and forage preferentially where caterpillar densities are the highest. Per capita predation, effects of birds on insects are often density‐dependent, rising with increasing available biomass, and plateauing at high prey densities (Atlegrim, 1989; Crawford & Jennings, 1989; Solomon, Glen, & Ashton, 1979), best described by a Type III functional response (Holling, 1959; Murdoch, 1973). We therefore expect that host plants showing the highest prey biomass will be the most valuable to songbirds, and if ungulate browse preference aligns with prey abundance across tree hosts, high browsing pressure will reduce overall prey availability for a community of songbirds.

**Figure 1:**
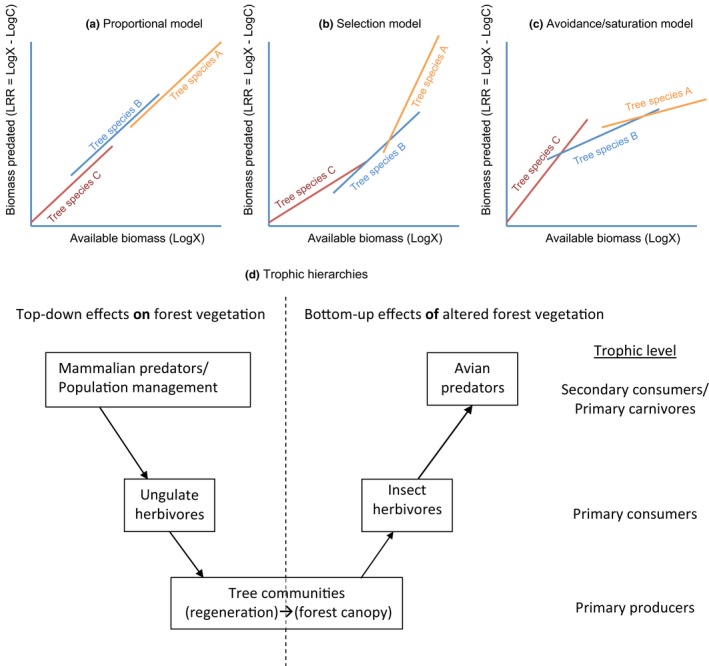
Alternative models for how caterpillar predation rates by birds may respond to increasing caterpillar density on different host tree species growing in the same community (a–c). In each model, tree species differ in their mean available caterpillar biomass (different range and mean on the x‐axis). In (a) the proportional model, birds forage on trees in proportion to the available caterpillar biomass; the per capita predation rate of caterpillars is constant across the range of caterpillar densities, and birds do not appear to be selecting or avoiding certain tree species depending on caterpillar density. In (b) the selection model, birds forage proportionally more on tree species that host higher caterpillar biomass; thus, the per capita rate of predation on caterpillars is higher (steeper slope) on tree species that host higher caterpillar densities. In (c) the avoidance/saturation model, birds forage proportionally less on tree species with higher caterpillar densities; thus, predation capacity seems to saturate on those species even though tree species in the same community with lower caterpillar biomass still have relatively high predation rates; this pattern may be due to low apparency of cryptic caterpillars, bird avoidance of certain caterpillar species, or prey inaccessibility due to plant architecture. (d) Trophic hierarchies that rely on tree communities, whereby altered browsing pressure may exert long‐term effects on birds (from Nuttle et al., 2011)

Here, we first test whether prey abundance predicts prey removal by birds by excluding birds from branches of six species of trees in northern hardwoods forests of Pennsylvania, USA. We know that tree species differ in mean caterpillar density (Butler & Strazanac, 2000; Futuyma & Gould, 1979; Karban & Ricklefs, 1983; Wheatall et al., 2013), and can be thereby ranked (x‐axes on Figure 1). We ask if overall predation tracks increase in prey density regardless of tree species (proportional model, Figure 1a), or if differences in prey density are associated with increases (selection model, Figure 1b) or decreases (avoidance/saturation model, Figure 1c) in predation rates across tree species. Departures from the proportional model suggest that total caterpillar biomass differs from accessible or acceptable prey biomass, for example, because birds forage more effectively on a particular host tree or prefer the prey found there (selection model), or avoid, cannot detect or capture, or cannot consume any more prey on a particular tree species (avoidance/saturation model). Second, we test whether changes in tree species composition due to white‐tailed deer (*Odocoileus virginianus*) browsing reduce prey availability for songbirds by aligning the value of trees species as sources of songbird prey with deer browse preferences. Finally, using data on tree species composition from plots exposed to experimentally controlled deer densities, we predict how changes in forest composition due to historic deer browsing may alter prey abundance for temperate forest songbirds.

## 2 MATERIALS AND METHODS

### 2.1 Overview

This study focuses on trophic interactions among broadleaf deciduous trees, phytophagous insects, and avian predators. Plant species studied include six broadleaf tree species of the Allegheny Hardwoods of northwestern Pennsylvania (Nuttle et al., 2011): black cherry (*Prunus serotina)*, pin cherry (*Prunus pensylvanica)*, American beech (*Fagus grandifolia)*, red maple (*Acer rubrum)*, sugar maple (*Acer saccharum*), and birch, represented by sweet birch and yellow birch (*Betula lenta* and *B. alleghaniensis),* respectively, because they cannot reliably be distinguished when young, they are grouped. Relative density of these species within Allegheny hardwoods forests varies depending on many factors, including deer density; collectively, they comprise over 98% of the basal area of canopy trees across deer density treatments reported in Nuttle et al. (2011). Many species of phytophagous larvae in the order Lepidoptera and in the hymenopteran suborder Symphyta (collectively referred to as caterpillars here) depend on these tree species. These larvae constitute the majority of the diet of migratory birds such as warblers (Parulidae) and vireos (Vireonidae) during the breeding season (Hagar, Dugger, & Starkey, 2007; Sample, Cooper, & Whitmore, 1993).

Sampling occurred during June and July of 2011 and 2012 at study sites (*N *= 10) located throughout Allegheny National Forest to coincide with the breeding and fledging period for these birds in this region. Using a paired treatment design, all trees chosen for the study had one branch exposed to and one branch protected from avian predation for the duration of the study. We used the difference between exposed and protected branches to evaluate avian predation response to prey density and host tree species identity (Mooney, Pratt, & Singer, 2012; Singer, Farkas, Skorik, & Mooney, 2012).

### 2.2 Sampling methods

We conducted avian exclusion studies in early and mid‐successional habitat generated within the past two decades by silviculture. A total of six sites were sampled each year, with two sites overlapping years, and four unique sites in each year. A site was operationally defined as a relatively homogeneous habitat with all study trees encompassed within a radius ≤0.75 km. Sites were located ≥3.5 km from one another to ensure reasonably independent caterpillar communities (Chen & Dorn, 2009; Kareiva, 1983; Mo, Baker, Keller, & Roush, 2001). Beginning in mid‐May of each year in each of three consecutive weeks, with each week starting a new “set,” we selected four individual sapling‐sized trees of each species. On each tree, we selected branch pairs of similar height (ca. 1.5 m above ground) and length (ranging from 0.75 m to 1.5 m). In rare cases when trees did not have two branches of approximately equal length and height from the ground, nearby trees were chosen. One branch randomly assigned as the treatment branch was enclosed in a mesh bag constructed with 1.9‐cm mesh Dalen Gardeneer DX‐7^®^ netting and secured approximately 50 cm from the tip of the branch with a small piece of plastic‐coated wire. The corresponding exposed branch was similarly marked to assure an approximately equivalent sample in each treatment in each pair. These netting materials have been used to successfully exclude birds from branches while allowing arthropod predators and parasites access similar to control branches (Böhm, Wells, & Kalko, 2011; Mooney, 2007; Singer et al., 2012). All tree species were present at all sites in 2011, resulting in 432 trees (864 individual branches) being sampled (*N *= 4 trees per species × 3 sets × 6 species × 6 sites). In 2012, *A. saccharum* was not found at one site and thus we sampled 420 branch pairs (840 individual branches) in 2012.

Prior to installing the nets, we removed all insect larvae from both the netted and nonnetted branches in each pair. During 2011 exclosure, netting remained on the same branches throughout the study period. Sampling effort in 2011, as quantified by mean leaf area of branches before treatment application in May, did not differ across treatments (ANOVA, *df* = 1, *F *= 0.033, *p *= .855), but because leaf damage increased throughout the summer on branches protected from birds, effective sampling effort decreased on protected branches throughout the season. In an attempt to prevent sampling effort discrepancies in 2012, we switched branch treatments by moving nets between sampling periods. Sampling effort in 2012, as quantified by mean leaf area sampled across all sampling periods, showed no significant changes with treatment (ANOVA, *df* = 1, *F *= 0.002, *p* = .968), indicating this modification kept the sampling effort consistent.

Each branch was sampled at four and eight weeks (during June and July) after initial treatment assignment in each year (2011 and 2012). During sampling, we inspected branches for larvae and placed them individually in 10‐ounce plastic deli containers with leaves of the host plant. All collected larvae were photographed, and their length was measured using an Olympus model SZ61 dissecting microscope with mounted Spot Idea^™^ USB camera and the SpotBasic software (Diagnostic Instruments, Inc.).

#### 2.2.1 Caterpillar biomass

During 2012, we weighed all collected larvae using an analytical balance with 0.0001‐g precision (Mettler Toledo PB153‐S/FACT Classic Plus model), and length‐weight regression equations were developed for Symphyta and the major Lepidoptera families represented in our collections. We also generated a general equation for all measured Lepidoptera and used this for unidentified caterpillars and those not belonging to a major family. We used these equations to estimate caterpillar biomass for both years. Larvae <5 mm and those belonging to the order Coleoptera were excluded from analysis. This cutoff in length corresponds roughly to observations that larvae smaller than 10–12 mg (corresponding minimum length of 6 mm) are often neglected or collected in low frequency by birds (Hagar et al., 2007; Naef‐Daenzer, 2000; Tinbergen, 1960).

During collection, the number of leaves on each branch was counted and assessed for chewing damage using a scale modified from that described by Martel and Maufette (1997). Each leaf was assigned a damage score, 0–1% (no damage), 1–10%, 10–25%, 25–50%, 50–75%, or >75%. A weighted average of the mid‐class values of damage for all leaves on a branch was used to calculate the overall percent intact score for each branch; leaves rated in the 0–1% category were considered 100% intact. This weighted average (percent intact score) was multiplied by the observed number of leaves to generate an adjusted number of intact leaves on each branch that accounts for reduction in mass due to damage. Average leaf mass was calculated using the dry mass of the most intact leaves to calculate average mass of one intact leaf for each species. Because we assessed damage of each branch during sampling and converted this into the number of intact leaves, we multiplied the average weight of one leaf by the adjusted number of intact leaves on each branch to obtain an estimate of leaf biomass of each branch.

To best connect changes in songbird prey with observed changes in foliage density in heavily browsed forests (Nuttle et al., 2011), we converted estimates of caterpillar biomass per unit leaf mass to caterpillar biomass per unit leaf area, based on estimates of specific leaf area. Leaf area was measured using scans of fresh leaves from the six tree species, sampled on 25 July 2012. Scanned electronic images were analyzed for leaf area using ImageJ software (Rasband, 2014). After scanning, leaves were placed in paper bags and dried at 80° F for 2 days, then weighed using an analytical balance with 0.0001‐g precision. Specific leaf area (g/cm^2^) was calculated from this sample and used to scale leaf mass to leaf area for each sample.

### 2.3 Data analysis

The number of larvae collected from any one branch was often very low or zero. Consequently, we pooled the four branch‐level values of caterpillar biomass by treatment within a tree species at a site for each sample event to compute caterpillar biomass per unit leaf area for each treatment by tree species by site combination.

#### 2.3.1 Caterpillar biomass differences across tree species and treatments

We tested for overall differences in caterpillar biomass per unit leaf area (g/m^2^) across tree species and treatment (exposed to or protected from birds) with a linear mixed model using week‐level groupings (sets) and site as random effects (*lmer* function in R 3.0.1) (Bates, Mächler, & Bolker, 2012). Tree species by treatment interaction terms were removed when nonsignificant. We conducted posthoc pairwise comparisons using least‐square means (*lsmeans* function in R 3.0.1). Due to known phenological differences in caterpillar communities and caterpillar sizes throughout the summer months in temperate forests (Futuyma & Gould, 1979; Summerville, Crist, & Science, 2003; Wheatall et al., 2013), we analyzed months separately. We analyzed years separately because of sampling differences described above.

#### 2.3.2 Effect of caterpillar biomass and tree species on avian predation

Avian predation, or removal of caterpillars by birds, was quantified as the log response ratio, LRR, of protected versus exposed branches. LRR is the log‐proportional change in mean caterpillar biomass between the treatment (exposed branches) and control (protected branches) and is used here to quantify an exclusion effect size (Hedges, Gurevitch, & Curtis, 1999; Singer et al., 2012). Thus, LRR = Log(*X* + *a*) − Log(*C* + *a*), where *X *= caterpillar biomass on protected branches (total biomass, not available to birds), *C *= caterpillar biomass on control branches (biomass left over after predation by birds), and *a* is a small number (0.1 g/m^2^, in the same order of magnitude as the lower range of biomass values) added to avoid taking the *Log* of zero.

We tested if LRR depended on total available caterpillar biomass on the protected branch (i.e., *Log*(*X + a)*), tree species, or their interaction using mixed models (*lmer* function). For this analysis, we summed biomass over treatment and month at each site and therefore included site as a random effect and analyzed months within years separately as noted above. We removed nonsignificant interaction effects from models unless their removal resulted in a poorer‐fitting model, as measured by an AIC value higher than that including the interaction term, in which instance we retained the model providing the lower AIC value (Bolker et al., 2009). The interaction term in our model provided a test of differences in density‐dependent response, which when significant indicated bird predation response to increasing prey density differed by tree species (as represented in Figure 1b,c). Comparisons of mean LRR across tree species provided a test of whether predation is comparable across tree species. We used the r.squaredGLMM function of the MuMIn package in R 3.1.1 to obtain estimates of model fit (Nakagawa & Schielzeth, 2013). Reported values are the conditional *R* ^2^ (*R* ^2^_C_), which include the random effects. Partial r‐square values for fixed effects were obtained as the change in *R* ^2^_C_ compared to a simplified model omitting the fixed effect of interest.

Finally, we tested if mean caterpillar biomass removed by birds or the scaled predation effect, LRR, is related to deer tree species preference. We based deer browsing preferences on Nuttle et al. (2011), which correspond well with those reported by Horsley et al. (2003). We ranked tree species according to their response (change in basal area) to increasing deer density. Regression analyses were conducted in R 3.0.1 using the linear model (lm) function.

#### 2.3.3 Predicted changes in caterpillar biomass due to historic deer browsing

The USDA Forest Service Northern Research Station maintained white‐tailed deer at a controlled, monitored range of densities for 10 years (1979–1990) in large enclosures in and near Allegheny National Forest (Tilghman, 1989) where our study was conducted. At each of four sites, four experimental deer density enclosures, 3.9, 7.8, 15.6, or 31.2 deer/km^2^, were established by adding radio‐collared deer. Ten percent of each enclosure was clear‐cut to simulate deer effects on regenerating forests, and permanent 400‐m^2^ sampling plots were established to monitor regeneration. The effects of forest management practices and deer density on tree species composition revealed by these plots are analyzed elsewhere (Horsley et al., 2003; Tilghman, 1989). We used data from these plots to predict how caterpillar biomass may shift with changes in tree species composition attributed largely to deer density. Using host tree basal area estimates from a 2010 tree composition study (unpublished data) and estimates of leaf area per unit basal area (Nuttle et al., 2011), we calculated stand‐level leaf area (m^2^/ha) for host tree species in the regeneration treatments at each deer density treatment at each site. Mean caterpillar biomass (g/m^2^ leaf area) removed by birds from host tree species (*X* – *C*) in June 2011 and 2012 was paired with stand‐scale leaf area estimates to generate estimated caterpillar removal from each species under different deer density regimes across the four sites. We tested the putative effect of deer density on caterpillar biomass across host species (deer density by tree species interaction) in this predictive analysis using a generalized linear mixed model with site as a random effect (*glmer* function in R 3.1.1) and the effect of deer density on stand‐level caterpillar biomass using a linear mixed model (*lmer* function in R 3.1.1)

## 3 RESULTS

### 3.1 Effect of bird exclusion on caterpillar biomass

**Figure 2:**
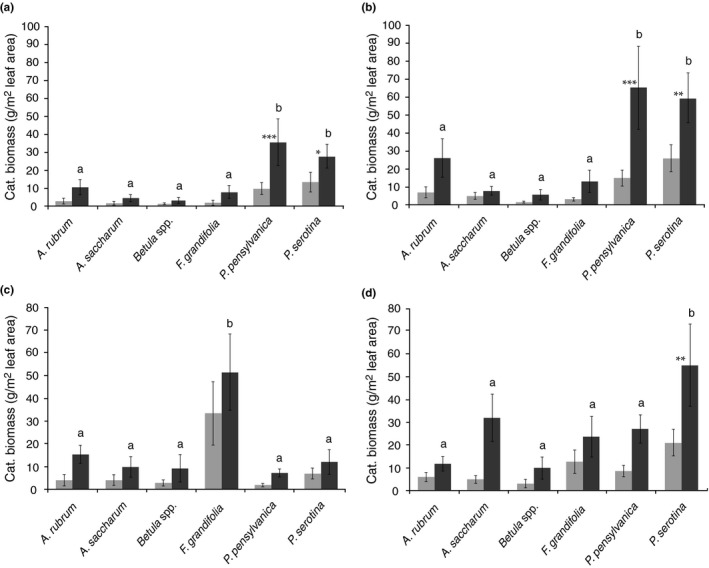
Mean (±1 SE) caterpillar biomass (g/m^2^) collected from tree branches exposed to (light gray) and protected from (dark gray) bird predation in June 2011 (a), July 2011 (b), June 2012 (c), and July 2012 (d). Letters indicate differences in larval biomass between protected branches across tree species, and asterisks indicate significant treatment effect within a species (* = *p* < .05, ** = *p* < .01, *** = *p* < .001)

In 2011, we collected 1707 caterpillars (703 from exposed branches, 1004 from protected branches). In 2012, we collected 1526 caterpillars (568 from exposed branches and 958 from protected branches). In both June and July 2011, *P. serotina* and *P. pensylvanica* supported significantly higher caterpillar biomass than other species (Figure 2a,b). Furthermore, only *Prunus* species showed significant increases in caterpillar biomass when protected from avian predators (Figure 2a,b). Findings differed in 2012, when during June only *F. grandifolia* showed significantly higher caterpillar biomass than other species and we did not detect bird effects on any tree species (Figure 2c). In July 2012, *P. serotina* again showed higher caterpillar biomass than other species and was the only tree species where bird exclusion resulted in increased caterpillar biomass (Figure 2d), though the effect of exclusion on two other species approached significance (*A. saccharum*,* p* = .052; *P. pensylvanica*,* p* = .059).

### 3.2 Role of caterpillar biomass and tree species on predation effects

**Figure 3:**
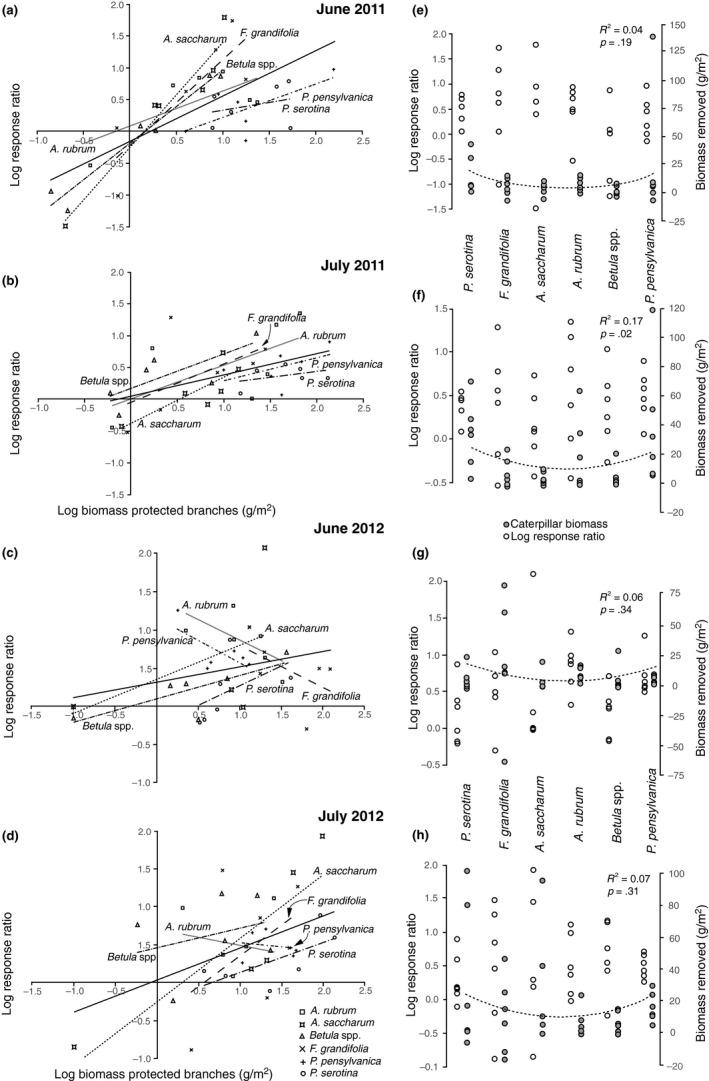
Changes in magnitude of bird effect over prey density and tree species during 2011 (June (a), July (b)) and 2012 (June (c), July (d)). Dotted lines indicate regression between LRR and the log of biomass on protected branches for individual tree species. Solid lines represent regression over all tree species. Regression plots (e‐h) depict quadratic fit between caterpillar biomass removed (filled circles, mean value at each site) and browse index of tree species based on their response (change in basal area) to increasing deer density based on Nuttle et al. (2011). Open circles depict Log Response Ratio values (all regressions nonsignificant)

Both available prey biomass and tree species showed an effect on avian predation response (as measured by the log response ratio, LRR) during all sample periods, but available prey biomass explained over twice the variance in avian predation response as tree species identity in all sampling periods except June 2012 (*R* ^2^_C_ in Table 1). Avian predation generally tracked increases in prey biomass (Figure 3a–d), but the effect of total prey biomass on predation rate differed by tree species during June both years (Table 1, interaction).

**Table 1:** Mixed‐effects model testing the contribution of prey biomass and foraging substrate (tree species) to prey removal by birds in June and July 2011 and 2012. Tests for each sampling period were conducted independently

	Overall model^a^	log(BiomassX)	Tree Species	Interaction b (log(BiomassX) × Tree Species)
June 2011				
*df*		1	5	5
*p*‐value		<.0001	<.001	.0077^b^
*R* ^2^_C_ c	0.7444	0.4736	0.1738	0.1017
July 2011				
*df*		1	5	–
*p*‐value		<.0001	.0248	–
*R* ^2^_C_ c	0.5594	0.4929	0.2341	–
June 2012				
*df*		1	5	5
*p*‐value		.0200	.0345	.1150 b
*R* ^2^_C_ c	0.5077	0.1736	0.2341	0.1071
July 2012				
*df*		1	5	–
*p*‐value		<.0001	.0056	–
*R* ^2^_C_	0.7158	0.6597	0.2603	–

a The *lmer* (lme4 package in R 3.1.1) function does not generate overall model *p*‐values or degrees of freedom.

b Retained if significant (*p* < .05) or if removing it significantly reduced overall model fit, as measured by a significant increase in model deviance.

c *R* ^2^ values from function r.squaredGLMM in the MuMIn package in R 3.1.1. Reported values are the conditional *R* ^2^, which include the random effects. Partial r‐square values for fixed effects (log(BiomassX), Tree Species, and Interaction) were obtained as the change in *R* ^2^_C_ compared to a simplified model omitting that fixed effect.

In June 2011, in line with the avoidance/saturation model, birds appeared to exhibit a weaker prey density‐dependent predation response on tree species with higher caterpillar biomass (shallower slopes of *Prunus* species, Figure 3a). The change in predation effect at high densities in June 2012 was more pronounced than in 2011, as avian predation effects on trees species with the highest ranked biomass, *F. grandifolia* and *A. rubrum*, exhibited negative density‐dependence (Figure 3c), a potential extension of the avoidance/saturation model. In June 2011, mean LRR decreased on tree species with increasing ranked biomass (Figure 3e), a trend not observed in June 2012 (Figure 3g), the only month when tree species identity explained more variation in bird response than available prey biomass (Table 1). Regardless of the biomass available on protected branches during June, *P. serotina* and *P. pensylvanica* fell below the overall mean LRR (solid lines in 3a–3d), indicating a saturation or avoidance by birds.

**Figure 4:**
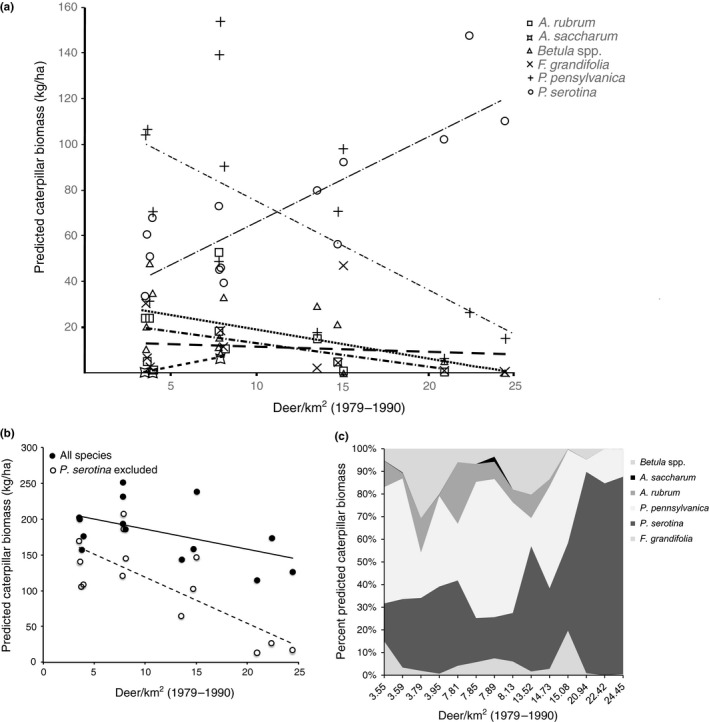
(a) Predicted prey biomass removed by birds across the six dominant tree species in Allegheny hardwoods forests as a function of deer density, as determined by branch‐level predation combined with stand‐level estimates of deer density effects on basal area of each tree species (deer density x tree species interaction term *p* < .0001). (b) Total prey biomass summed across all species (closed circles, solid line; *p* = .273, *R* ^2^_C_ = 0.286). The removal of *Prunus serotina* results in predicted significant decrease in prey biomass from other tree species at high historic deer density exposure (open circles, dotted line; *p* < .0001, *R* ^2^_C_ = 0.62). (c) Shifts in biomass distributions at historic exposure to different deer densities

In July of both years, available caterpillar biomass explained more than twice the variation in avian predation response and the rate at which birds removed prey biomass was comparable across tree species (no interaction; Table 1, Figures 3 and 4), in line with the proportional removal model (Figure 1a). Though prey removal was comparable across tree species, mean predation response did differ in both years, generally decreasing with increasing rank available biomass (Table 1, Figure 3f,h) and was lowest on *P. serotina*. Thus, the bird response to increases in prey density on *P. serotina* was comparable with other tree species, but as in June, *P. serotina* fell significantly below others in mean bird effects, again supporting the avoidance/saturation model.

Predation effects did not show a linear correlation with deer browse preference (Figure 3e–h). Prey biomass removed did show a significant, quadratic relationship with deer browse preference over all sampling periods (mixed‐effects model, *df* = 1, *p* = .007). This trend was driven primarily by high prey biomass removal from both *Prunus* species and did not exist within three of four sampling periods when evaluated separately (Figure 3e,g,h).

### 3.3 Predicted effect of deer density on stand‐level prey availability

Our predictive analysis indicated the response of stand‐level prey availability to deer density varies by tree species (Figure 4a; interaction term, deer density by tree species, *p* < .0001), due largely to the underlying shifts in tree species composition with increased deer density. The combined effect of increased representation of *P. serotina* in plots exposed to high deer densities, and high prey biomass available per unit leaf area on this species resulted in a predicted high prey biomass removal from *P. serotina* at higher deer densities. *P. pensylvanica* supports relatively high prey biomass per unit leaf area, but precipitous declines in *P. pensylvanica* presence in stands exposed to high deer density are predicted to decrease stand‐level prey available from *P. pensylvanica* (Figure 4c). Deer density did not affect projected stand‐level prey availability (*p* = .273, Figure 4b). This appears to be a kind of compensatory effect driven by *P. serotina*, as its removal from the model resulted in a significant deer density effect on caterpillar biomass (*p* < .0001, Figure 4b). The distribution of stand‐level prey biomass across tree species tracks changes in tree community composition; therefore, at the highest deer densities prey biomass is predicted to come predominantly from *P. serotina* (Figure 4c).

## 4 DISCUSSION

Although tree species differ in caterpillar productivity and bird predation on caterpillars, in line with existing evidence for density‐dependent avian predation on caterpillars, differences in estimated predation within and across tree species were explained largely by variation in available prey biomass. There existed a season effect in this density‐dependence; during June of both years (Figure 3a,c), trends reflect predictions of the avoidance/saturation model (Figure 1c) wherein tree species with higher densities of caterpillars generally were associated with weaker or negative density‐dependent predation effects (shallow or negative slopes). During July of both years (Figure 3b,d), the proportional removal model (Figure 1a), wherein increase in predation track increases available prey items, better describes the bird predation response. In both 2011 and 2012, tree species rankings according to relative caterpillar biomass were similar whether based on exposed or protected branches, and thus inferences about relative caterpillar productivity derived from exposed branches (as in Nuttle et al., 2011; Wheatall et al., 2013, and other studies of caterpillar communities) appear valid.

Forests of Northwestern Pennsylvania exposed to high white‐tailed deer densities regenerate to near‐monocultures of *P. serotina* (Horsley et al., 2003) and show reductions in understory plant diversity decades after deer densities are reduced to presettlement levels (Nuttle et al., 2014; Redding, 1995). *Prunus* species show significantly higher caterpillar abundance than co‐occurring hardwood species (e.g., *A. rubrum, A. pensylvanicum., Betula* spp*., F. grandifolia*; Figure 2, see also Singer et al., 2012; Wheatall et al., 2013) but experience opposite deer browsing pressure— *P. serotina* is generally avoided, whereas *P. pensylvanica* is favored over co‐occurring hardwoods.

Nuttle et al. (2011) found relatively low numbers of caterpillars on *P. serotina* (avoided by deer) compared to *P. pensylvanica* (preferred by deer) and hypothesized that deer‐induced changes to primary producer communities may lead to reductions in food availability for migratory birds, reducing bird density in forests affected by high deer density. In a follow‐up study, Wheatall et al. (2013) reported similar host‐caterpillar density rankings as Nuttle et al. (2011), though differences in caterpillar density between *P. pensylvanica* and *P. serotina* were not as pronounced. Data presented here show that protected branches of both *Prunus* species ranked highest in caterpillar density, and although generally birds removed more caterpillars from both species than other trees, bird predation on *P. serotina* was saturated or otherwise limited at high caterpillar densities (Figure 3). In three of four months studied, *P. serotina* and *P*. *pensylvanica* showed the highest available caterpillar biomass, but the community of avian predators was often unable to fully capitalize on this biomass as indicated by a lower mean LRR than on tree species host to lesser biomass (Figure 3). Spanning across all tree species, the distribution of data points for June suggests an overall saturation pattern in bird response to increasing prey biomass (Figure 3a,c). This same pattern could indicate avoidance rather than saturation, as measurements of biomass on different hosts are concomitant; that is, birds are exhibiting lower density‐dependent predation on certain hosts (*A. rubrum*,* F. grandifolia*,* P. pensylvanica*) than others at the same time and location. Predation across the whole forest community is therefore not saturated, suggesting birds are selectively foraging at the tree species scale, either because some prey remain undetectable or are unacceptable to birds.

Unlike in June, in July of both years, predation response to increasing prey biomass was comparable across tree species (no interaction, Table 1). Bird preferences change with changes in caterpillar community composition throughout the season (Naef‐Daenzer, 2000; Royama, 1970), and food demands are expected to increase with recruitment in bird populations by young, naive foragers. Nevertheless, though total predation was high (Figure 2), *P. serotina* still experienced the lowest mean predation rates (LRR; Figure 3f,h; Table 2): Bird response to increasing prey biomass (slope) on *P. serotina* was comparable to that of other species, but the mean bird effect (intercept and mean LRR) was shifted down. This suggests that while bird response to incremental increases in prey biomass on *P. serotina* is comparable to other tree species, there is a group of prey items birds generally avoid or cannot detect, but which we collected. *P. serotina* is known to support two species of social caterpillars generally avoided by birds, the eastern tent caterpillar (*Malacosoma americanum*) (Fitzgerald, 1995) and the fall webworm (*Hyphantria cunea*) (Morris, 1972), but both were rare to absent from our collections and did not contribute to this effect. Perhaps, there are other species of caterpillars abundant on *P. serotina* and avoided by birds; further analysis of shifts in caterpillar community composition caused by avian predation will provide insight into mechanism, specifically whether particular caterpillar species are missed or avoided by birds as has been found in other exclusion studies (Atlegrim, 1989).

**Table 2:** Mean predation rate (LRR = log response ratio), and caterpillar biomass removed per unit leaf area during each sample period. Tree species are in order from most browse avoided (*Prunus serotina*) to most browse preferred (*Prunus pensylvanica*; Nuttle et al., 2011)

Tree species	Sample period	Mean LRR	Mean biomass removed (g/cm^2^)
*Prunus serotina*	June 2011	0.412	14.14
	July 2011	0.367	33.40
	June 2012	0.188	5.08
	July 2012	0.302	33.81
*Fagus grandifolia*	June 2011	0.584	5.94
	July 2011	0.363	9.88
	June 2012	0.476	17.97
	July 2012	0.495	10.90
*Acer saccharum*	June 2011	0.456	2.94
	July 2011	0.148	2.71
	June 2012	0.461	5.74
	July 2012	0.602	28.86
*Acer rubrum*	June 2011	0.489	7.77
	July 2011	0.542	18.86
	June 2012	0.841	11.38
	July 2012	0.502	5.85
*Betula* sp.	June 2011	−0.053	1.85
	July 2011	0.363	4.14
	June 2012	0.220	6.39
	July 2012	0.638	6.83
*Prunus pensylvanica*	June 2011	0.341	25.69
	July 2011	0.504	49.98
	June 2012	0.705	5.36
	July 2012	0.492	18.41

Deer prefer to browse *Prunus pensylvanica* and thereby eliminate it from some forests (Jordan, 1967). Generally, our data show that *P. pensylvanica* provides caterpillar biomass comparable to that of *P. serotina* (Figure 2), which deer avoid. In 2011, *P. pensylvanica* provided significantly more caterpillar biomass for birds than any other tree species (Figure 2). Coupled with the observation that leaf area per unit tree basal area is significantly lower on *P. serotina* compared to *P. pensylvanica* (Nuttle et al., 2011), we predicted the replacement of *P. pensylvanica* by *P. serotina* by deer overbrowsing may thus eliminate a substantial proportion of food for birds. We did not find support for this and instead found that predicted total biomass at the stand scale is not affected by deer density, an effect driven by *P. serotina*, as increasing abundance of this species offsets losses from the other species (Figure 4b,c). Therefore, biomass predated from particular host trees does change with increasing deer browsing pressure; tracking changes in forest composition, at low deer densities prey biomass, is more evenly distributed across tree species, whereas at high deer densities the majority of prey biomass hails from black cherry (Figure 4c).

Here, we predict that high caterpillar density on *P. serotina* will compensate for the lower foliage density reported by Nuttle et al., 2011, bringing caterpillar availability in black‐cherry‐dominated stands up to that in more diverse forests. However, this prediction does not take into account differences in mean predation *response* across *Prunus* species. We assessed predation rates on caterpillars in regenerating forest communities composed of a diverse mix of tree species, and these results may not scale to communities dominated by any one tree species. It will be crucial to study if forests exposed to high deer browsing pressure that regenerate to near‐monocultures of *P. serotina* also experience reduced bird predation effects on their caterpillar biomass.

In summary, avian predation effects are largely explained by differences in caterpillar productivity across tree species, consistent with the avoidance/saturation conceptual model (Figure 1c) and traditional density‐dependent foraging models (Holling, 1959; Murdoch, 1973). Tree species identity influenced per capita caterpillar removal by birds because tree species differ in their overall productivity as a substrate for caterpillars and because avian predation was less effective at particularly high caterpillar densities, which occurred most often on *Prunus* species. Because we investigated mixed species communities, these data do not support that bird populations were numerically saturated, but that caterpillar removal on highly productive hosts was functionally saturated. Our results bring into question why birds leave abundant populations of caterpillars on *P. serotina*, while removing a higher proportion of caterpillar biomass on neighboring trees with lower prey availability, an effect potentially due to differences in foraging preferences across bird species or unpalatability (Müller et al., 2006) or crypsis (Lichter‐Marck et al., 2015) of caterpillars thriving on *P. serotina*. This result may also have important implications for forest pest management, as near‐monocultures of *P. serotina* species may support high densities of caterpillars not effectively controlled by avian predation.

## CONFLICT OF INTEREST

The authors declare that they have no conflict of interest.

## AUTHOR CONTRIBUTIONS

RKG, EHY, and TJN conceived and designed the experiments and wrote the manuscript. RKG and EHY collected the data. RKG and TJN analyzed the data.

## DATA AVAILABILITY

The datasets from the current study available from the corresponding author on reasonable request.
