# Supplementary figures and images for: Opposing deer and caterpillar foraging preferences may prevent reductions in songbird prey biomass in historically overbrowsed forests
Source: Ecol Evol. 2017 Dec 2;8(1):560–71. doi: 10.1002/ece3.3497 (PMC5756885; doi:10.1002/ece3.3497)

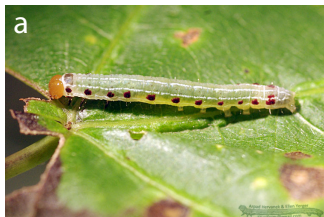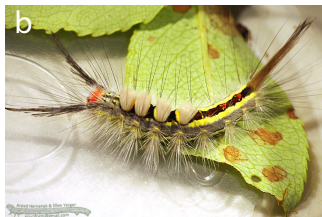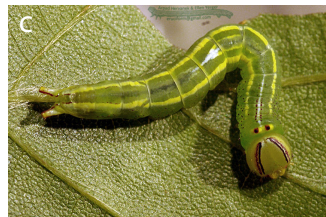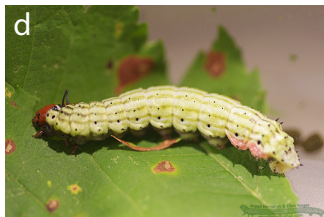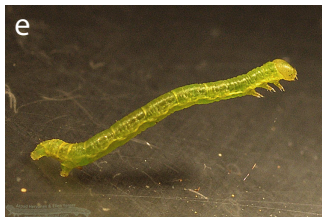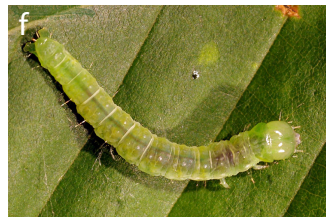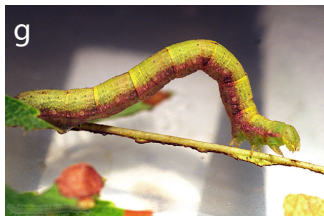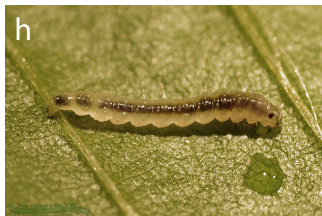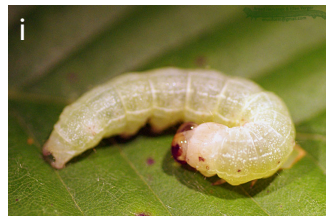

Supplement: Supplementary file 1 [file ECE3-8-560-s001.pdf]
